# Cyproheptadine Enhances the *I*_K_ of Mouse Cortical Neurons through Sigma-1 Receptor-Mediated Intracellular Signal Pathway

**DOI:** 10.1371/journal.pone.0041303

**Published:** 2012-07-23

**Authors:** Yan-Lin He, Chun-Lei Zhang, Xiao-Fei Gao, Jin-Jing Yao, Chang-Long Hu, Yan-Ai Mei

**Affiliations:** Institutes of Brain Science, School of Life Sciences and State Key Laboratory of Medical Neurobiology, Fudan University, Shanghai, China; Chiba University Center for Forensic Mental Health, Japan

## Abstract

Cyproheptadine (CPH) is a histamine- and serotonin-receptor antagonist, and its effects are observed recently in the modulation of multiple intracellular signals. In this study, we used cortical neurons and HEK-293 cells transfected with Kv2.1 α-subunit to address whether CPH modify neural voltage-gated K^+^ channels by a mechanism independent of its serotonergic and histaminergic properties. Our results demonstrate that intracellularly delivered CPH increased the *I*_K_ by reducing the activity of protein kinas A (PKA). Inhibition of G_i_ eliminated the CPH-induced effect on both the *I*_K_ and PKA. Blocking of 5-HT-, M-, D_2_-, H_1_- or H_2_- type GPCR receptors with relevant antagonists did not eliminate the CPH-induced effect on the *I*_K_. Antagonists of the sigma-1 receptor, however, blocked the effect of CPH. Moreover, the inhibition of sigma-1 by siRNA knockdown significantly reduced the CPH-induced effect on the *I*_K_. On the contrary, sigma-1 receptor agonist mimicked the effects of CPH on the induction of *I*_K_. A ligand-receptor binding assay indicated that CPH bound to the sigma-1 receptor. Similar effect of CPH were obtained from HEK-293 cells transfected with the α-subunit of Kv2.1. In overall, we reveal for the first time that CPH enhances the *I*_K_ by modulating activity of PKA, and that the associated activation of the sigma-1 receptor/G_i_-protein pathway might be involved. Our findings illustrate an uncharacterized effect of CPH on neuron excitability through the *I*_K_, which is independent of histamine H_1_ and serotonin receptors.

## Introduction

Cyproheptadine (CPH) is typically used as an antihistamine. CPH is also widely applicable as an H_1_ and 5-hydroxytryptamine (5-HT) receptor antagonist for the treatment of allergic reactions, as a prophylactic for migraines and in the symptomatic treatment of metastatic carcinoid syndrome [1]. As a first-generation antihistamine, CPH produces side effects such as sedation and reduced cognitive function [2]. Moreover, CPH is thought to resemble clozapine, which is an atypical antipsychotic that is used to treat schizophrenia [3]. Interestingly, the structure of CPH is similar to that of antidepressants such as amitriptyline, imipramine and N-methylamitriptyline [4]. These data suggest that CPH might play a role in the central nervous system (CNS). However, few studies have explored the cellular or molecular mechanisms by which CPH acts in the CNS or in primary neurons.

CPH possesses multiple pharmacological activities and acts as antagonist for serotonergic, dopaminergic, histaminergic, adrenergic or muscarinic receptors due to its relatively high binding affinity [5]. In addition to the previously mentioned receptor-dependent effects, CPH also directly inhibits ion channels, such as potassium (K^+^), sodium (Na^+^) and N-type and L-type calcium (Ca^2+^) channels in cardiac cells [6]. In addition, it has recently been reported that CPH induces apoptosis in leukemia cells, which is independent of its known activity as a histamine and serotonin receptor antagonist [7]. Similarly, the pancreatic β-cell toxicity that results from CPH exposure appears to be unrelated to its serotonergic or histaminergic properties because pharmacologically inactive metabolites of CPH also exhibit the same toxicity [8]. Moreover, recent studies have suggested that the diabetogenic effect of CPH may be at least partially due to altered regulation of protein synthesis [9]. These data indicate CPH effect independent of serotonergic or histaminergic receptors. Perhaps, CPH may act as an intracellular signaling molecule by binding to intracellular sites, such as one reported on sigma-1 receptor [10]. Notably, previous studies have indicated that sigma-1 receptor is widely distributed in CNS neurons and is associated with multiple intracellular signaling pathways [11]. Therefore, the serotonergic or histaminergic receptor independent effects mediated by CPH may be channeled, directly or indirectly, by targeting the sigma-1 receptor.

Among the voltage-gated ion channels, K^+^ channels are extremely diverse in structure and function and are one of the most important signaling macromolecules in both neuronal and non-neuronal cells. In the CNS, the activity of K^+^ channels determines the frequency and duration of action potentials [12]. Modifying K^+^ channels can alter neuronal excitability and modulating neurotransmitter release [13]. Inhibitors and activators of K^+^ channels have been proposed as potential therapies for a diverse array of neurological and cardiovascular disorders including stroke, hypertension, mental illness and others [14]. Although CPH has previously been reported to directly inhibit ion channels, few studies have addressed the possibility that CPH modifies the activity of neuronal voltage-gated K^+^ (Kv) channels by a mechanism unrelated to its serotonergic and histaminergic properties.

In this study, we investigated the effects of CPH on the delayed rectifier outward potassium current (*I*_K_) in primary cultures of cortical neurons using the whole-cell patch-clamp technique. The *I*_K_ was significantly enhanced by the intracellular application of CPH through its modulation of the sigma-1 receptor via cAMP/PKA signaling to specifically regulate Kv2.1 α-subunit.

## Results

### Both the Intracellular and Extracellular Application of Cyproheptadine Enhance the *I*_K_

**Figure 1:**
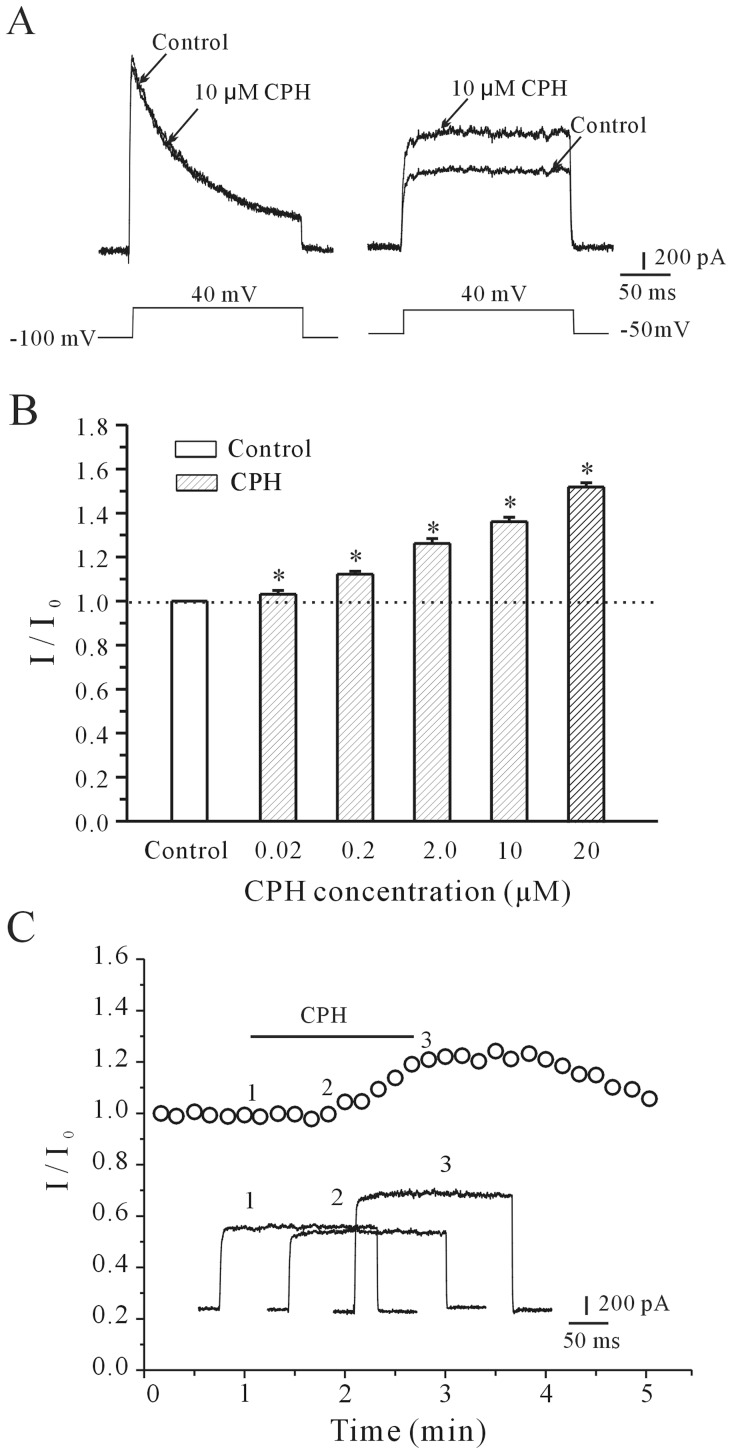
Both the intracellular and extracellular application of CPH enhances the *I*_K_ in a concentration-dependent manner. (A) *I*_A_ and *I*_K_ traces recorded intracellularly in the absence and presence of 10 µM cyproheptadine (CPH). The *I*_A_ was obtained by the application of depolarizing pulses to +40 mV from a holding potential of −100 mV, and the *I*_K_ currents were evoked with 200 ms depolarizing pulses to +40 mV from a holding potential of −50 mV. (B) A statistical analysis of the concentration-dependent effect of CPH on the *I*_K_ values. The data are reported as the mean ± S.E.M. from 6–10 cells. *P<0.05 when comparing different concentrations of CPH by a one-way ANOVA. (C) The time-course of the *I*_K_ changes induced by the extracellular application of 10 µM CPH. The insets in the graphs show superimposed *I*_K_ traces from the initial control levels (after the establishment of the whole-cell configuration) and after the external infusion of CPH. The time points (1, 2, and 3) noted on the curves correspond to the superimposed *I*_K_ traces illustrated by the insets.

Cortical neurons display two main voltage-dependent outward K^+^ currents, the delayed rectifier potassium current (*I*_K_) and the transient potassium current (*I*_A_), which can be specifically blocked by 20 mM TEA and 5 mM 4-AP, respectively. We first determined whether CPH affected either the *I*_A_ or the *I*_K_ using corresponding voltage protocols and specific K^+^ channel blockers. In the presence of 5 mM TEA, the *I*_A_ was obtained by depolarizing pulses to +40 mV from a holding potential of −100 mV, and the intracellular application of CPH did not affect the *I*_A_ (Fig. 1A, left). When 4-AP was added to the bath solution, the *I*_K_ was evoked by 200 ms of depolarization to +40 mV from the holding potential of −50 mV, and the intracellular application of CPH significantly enhanced the *I*_K_ (Fig. 1A, right). These data suggest that CPH selectively modulates the *I*_K_.

CPH is a lipid-soluble and membrane-permeable molecule, and its effect as a H_1_ and serotonin receptor antagonist has recently been reported [7]. We first examined the intracellular effect by the addition of CPH via a micropipette. The *I*_K_ of cortical neurons reached its maximum amplitude within 30–60 seconds. This CPH-induced effect on the *I*_K_ was concentration-dependent from 20 nM to 20 µM of CPH. The increases in the *I*_K_ induced by 20 nM, 200 nM, 2 µM, 10 µM and 20 µM of CPH were 3.1±1.1% (*n* = 7), 12.21±1.37% (*n* = 8), 26.16±2.19% (*n* = 7), 36.1±2.01% (*n* = 9) and 52.48±1.91% (*n* = 8), respectively. The statistical analysis of these concentration-response data is presented in Fig. 1B. We also tested whether the extracellular application of CPH affected the *I*_K_. The results showed that applying CPH by extracellular perfusion induced an increase in the *I*_K_ that was similar to the intracellular application. However, the maximum effect was observed after 3 min (Fig. 1C). Therefore, to obtain a rapid and stable effect, a CPH solution was applied intracellularly using a micropipette in the following electrophysiology experiment.

**Figure 2:**
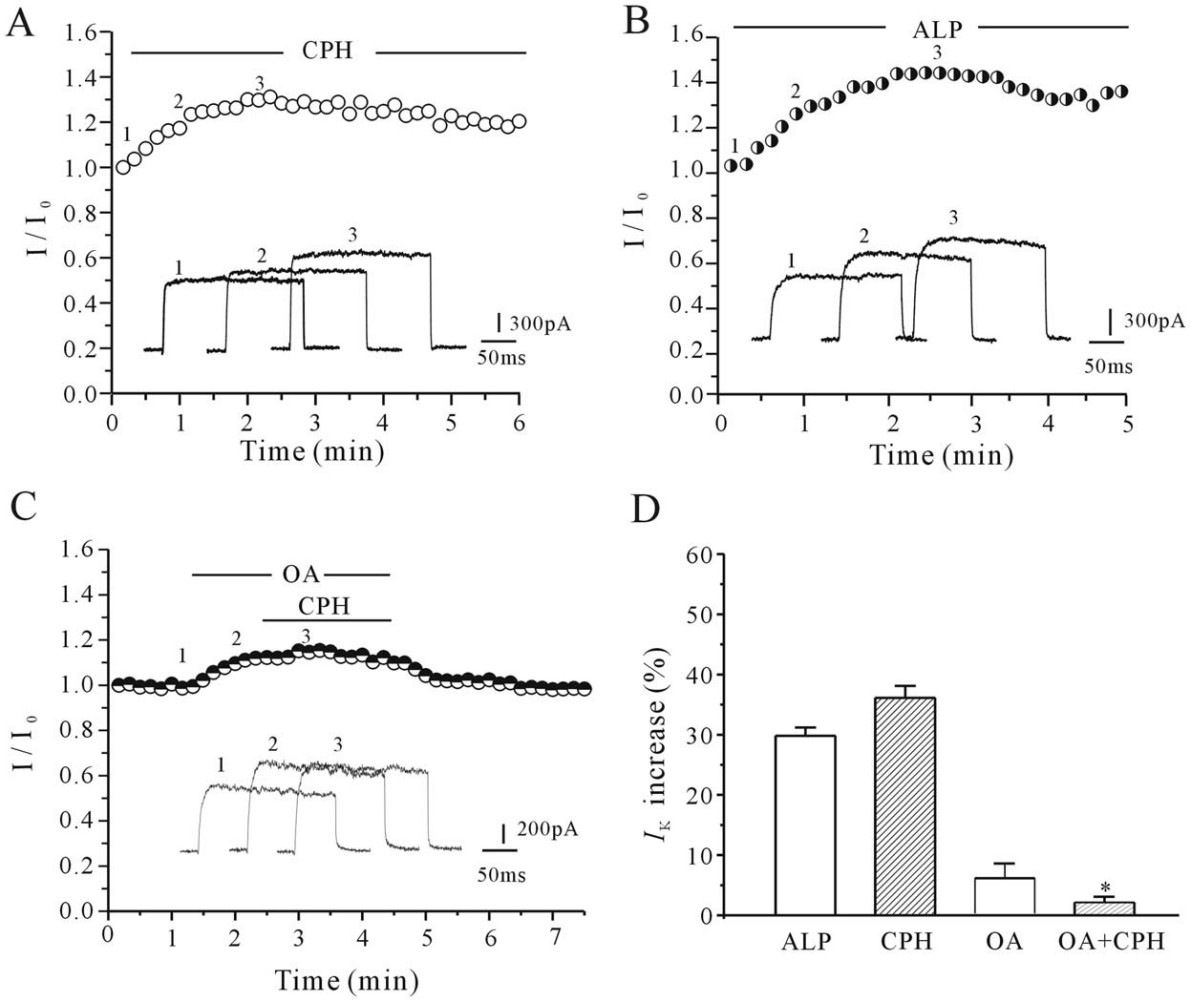
The effect of alkaline phosphatase and its inhibitor on the CPH-induced activation of the *I*_K_. (A) The time-course of the *I*_K_ amplitude changes induced by an intracellular application of 10 µM CPH. (B) The application of alkaline phosphatase (ALP, 200 u/L) mimicked the CPH-induced increase in the *I*_K_. (C) The application of PP1 inhibitor (okadaic acid, 200 nM) reduced the CPH-induced increase in the *I*_K_. (D) A statistical analysis of these results. The data are reported as the mean ± S.E.M. from 6 cells. *P<0.05 when compared with CPH and tetramisole.

Because the *I*_K_ increase can be modified by membrane channel protein dephosphorylation [15], alkaline phosphatase (ALP) was used to investigate whether the effect of CPH on the *I*_K_ might be due to a CPH-induced dephosphorylation of the *I*_K_ channel. As shown in Fig. 2B, ALP (200 unit/L) alone increased the *I*_K_ by 29.8±1.4% (*n* = 6), a result similar with CPH shown in Fig. 2A. Previous studies suggested that protein phosphatase 1 (PP1) contributes to the maintenance of constitutive phosphorylation of Kv2.1 subunit of *I*_K_ channel in cortical neurons [16]. We used okadaic acid, the PP1 inhibitor, to test this hypothesis. The result presented in Fig. 2C showed that okadaic acid (200 nM) alone increased the *I*_K_ by 4.92±2.0% (*n = *6), and in the presence of okadaic acid the 10 µM CPH-induced increase in the *I*_K_ was significantly reduced from 36.1±2.01% (CPH alone) to 4.59±2.0% (*n = *6). A statistical analysis of these data (Fig. 2D) suggested that the effect of CPH on the *I*_K_ was not direct but might be instead mediated by decreasing K^+^ channel phosphorylation.

### The cAMP/PKA Pathway Contributes to the Cyproheptadine-mediated I_K_ Increase

**Figure 3:**
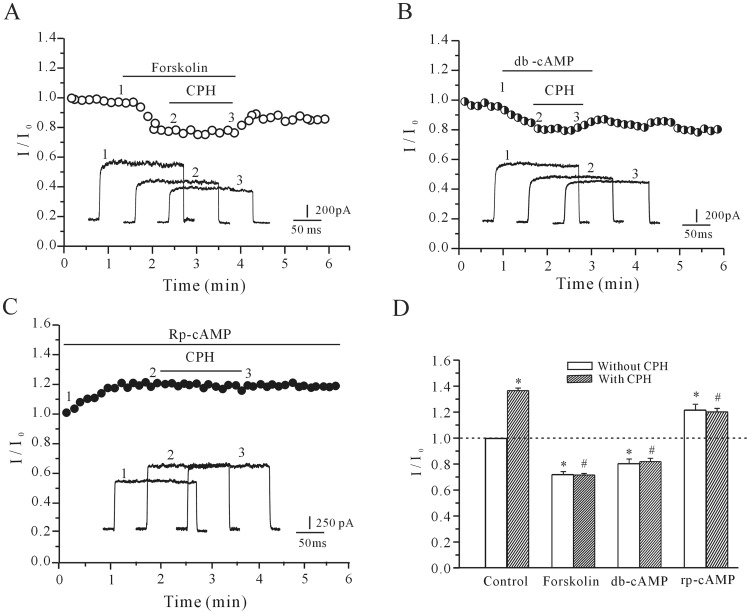
The effects of cAMP/PKA pathway activation or inhibition on the CPH-induced enhancement of the *I*_K_. (A and B) The time course of the *I*_K_ changes induced by the intracellular application of 10 µM CPH in the presence of 20 µM forskolin (A) or 20 µM db-cAMP (B). (C) The application of 20 µM Rp-cAMP mimicked the CPH-induced increase of the *I*_K_. (D) A statistical analysis of the effects of forskolin, db-cAMP and Rp-cAMP on the CPH-induced *I*_K_ increase. The data are reported as the mean ± S.E.M. from 7–9 cells. *P<0.05 when compared with the control;^ #^ *P*<0.05 when compared with CPH alone.

Because the PKA pathway plays an important role in channel phosphorylation, the effects of CPH were studied in the presence of forskolin, dibutyryl cAMP (db-cAMP) and Rp-cAMP to address whether the cAMP/PKA pathway contributed to the CPH-mediated increase of the *I*_K_. Forskolin or db-cAMP was applied to the bath solution to activate adenylate cyclase or PKA, respectively. Perfusion of cortical neuron with 10 µM forskolin provoked a gradual decrease in *I*_K_ amplitude by 22.9±3.8% (*n* = 5), and in the presence of 10 µM forskolin, CPH increased the *I*_K_ amplitude by only 4.9±1.0% (*n* = 8) (Fig. 3A), which is significantly less than CPH alone (36.1±2.01%). The administration of db-cAMP also eliminated the effect of CPH on the *I*_K_ (Fig. 3B). A uniform perfusion of 20 µM db-cAMP, produced a significant reduction of 23.4±2.1% in *I*_K_ currents, and abolished the CPH-induced inhibitory effect on *I*_K_. In the presence of 20 µM db-cAMP, CPH only increased the *I*_K_ by 4.3±0.9% (*n = *7), which is similar to the forskolin results. However, the application of Rp-cAMP, a membrane-permeable specific PKA inhibitor, mimicked the effect of CPH and increased the *I*_K_ amplitude by 24.7±2.32% (*n = *5). In the presence of both Rp-cAMP and 10 µM CPH, the *I*_K_ was further increased only slightly by 2.8±0.6% (*n = *8) relative to the baseline levels (Fig. 3C). Taken together, these results indicate critical contribution of the cAMP/PKA pathway in the CPH-induced *I*_K_ increase. A statistical analysis of these data is shown in Fig. 3D.

**Figure 4:**
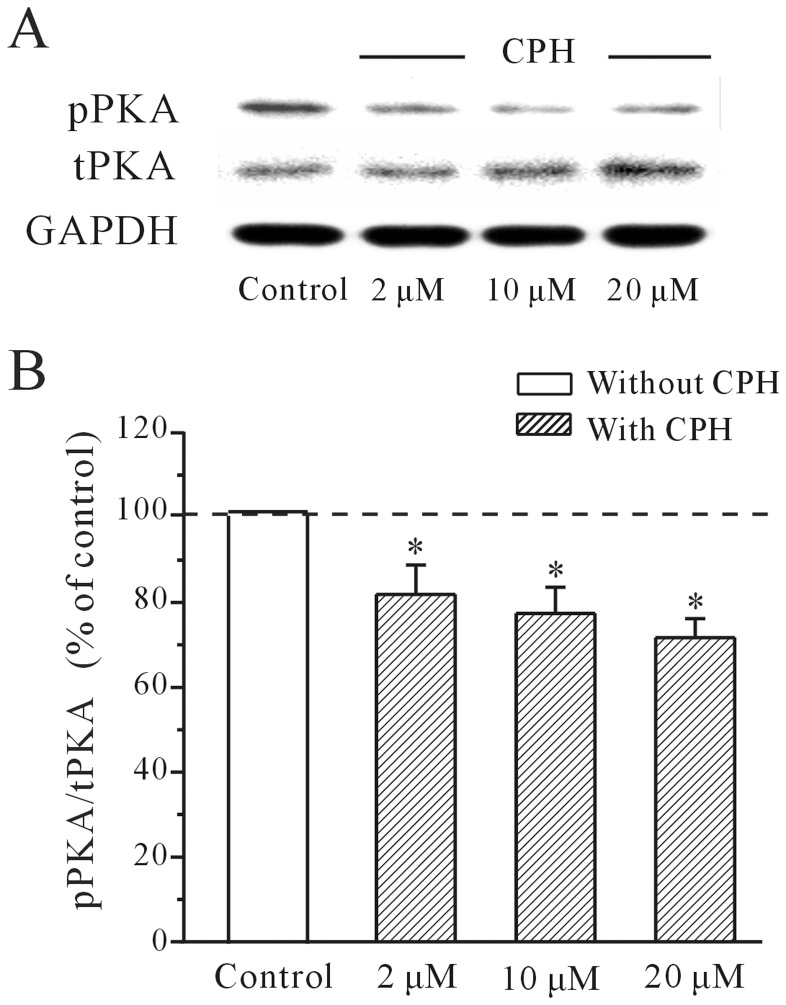
CPH inhibits the intracellular levels of phosphorylated PKA in mouse cortical neurons. (A) The levels of total PKA (tPKA) and phosphorylated PKA (pPKA) levels in response to CPH were detected by Western immunoblotting. (B) A quantitative statistical analysis of the pPKA activity was performed. *P<0.05 when comparing multiple concentrations of CPH by a one-way ANOVA. The data are reported as the mean ± S.E.M. obtained from 5 independent experiments.

Next, western blot was used to measure the levels of phosphorylated PKA (pPKA) in response to CPH application. The results demonstrated that there was a significant and dose-dependent decrease in the pPKA levels of cortical neurons that were incubated with CPH for 5 min (Fig. 4A). In the presence of 2 µM, 10 µM and 20 µM CPH, the activity of pPKA were significantly decreased to 81.3±6.9%, 76.5±6.1%, and 71.2±4.4% of the control values (*n = *4), respectively. The results of the quantitative statistical analysis of the pPKA activity are presented in Fig. 4B.

### CPH Targets G_i_ Protein-coupled Receptor Signaling Through Sigma-1 Receptor

**Figure 5:**
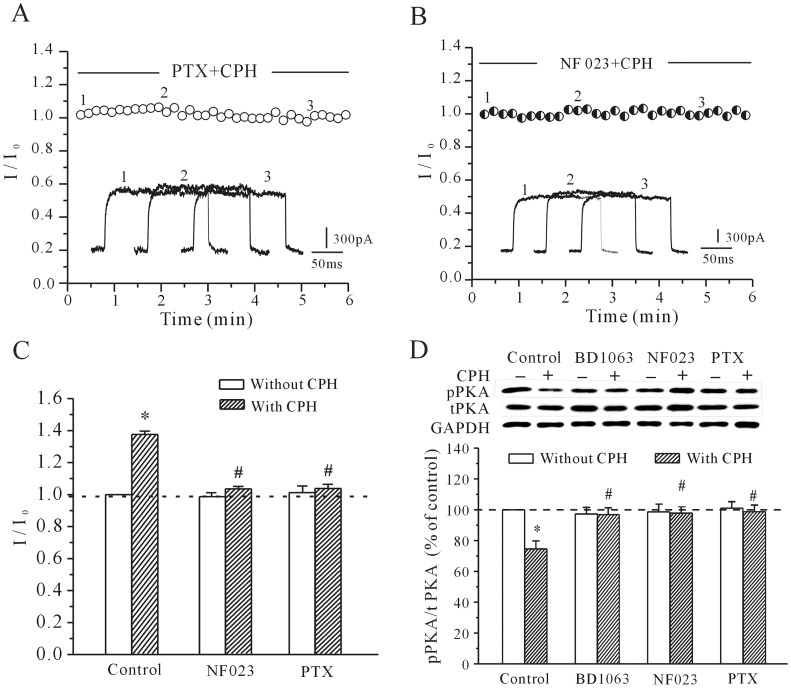
The effects of PTX and NF023 on the CPH-induced increase of the *I*_K_ and the decrease of the pPKA levels. (A and B) The time course of the *I*_K_ changes induced by CPH in cells pre-incubated with 200 ng/ml PTX or in the presence of 20 µM NF023. (C) A quantitative statistical analysis of the effects of PTX and NF023 on the CPH-induced *I*_K_ activation. The data are reported as the mean ± S.E.M. from 7 cells. *P<0.05 when compared with the control; ^#^ *P*<0.05 when compared with CPH alone. (D) PTX and NF023 both reversed the CPH**-**induced decrease in pPKA levels. The data are reported as the mean ± S.E.M. from 6 different experiments. *P<0.05 when compared with the control; ^#^ *P*<0.05 when compared with CPH alone.

G_i_ negatively couples with the cAMP/PKA pathway; therefore, selective blockers of G_i_ were used to determine whether the G_i_ protein was associated with the CPH-induced increase in *I*_K_ and the decrease in PKA activity in cortical neurons. Our results indicated that pre-incubation of cortical neurons with selective blockers of G_i_ (pertussis toxin (PTX) and NF023) significantly attenuated the CPH-induced increase in the *I*_K_ (Figs. 5A and 5B). 200 ng/ml PTX and 20 µM NF023 alone did not alter the *I*_K_ amplitude (Fig. 5C). After the cortical neurons were incubated with either 200 ng/ml PTX for 2 hours or 20 µM NF023 for 5 min, the addition of 10 µM CPH only increased the *I*_K_ by 7.31±0.77% (*n* = 9) and 3.73±0.84% (*n* = 7), respectively. These values were significantly different from those obtained with CPH alone (36.1±2.01%, Fig. 5C). In addition to affecting the *I*_K_ recordings, blocking G_i_ with PTX and NF023 also reduced the CPH-induced inhibitory effect on the intracellular pPKA levels. After the cortical neurons were incubated with either 200 ng/ml PTX for 2 hours or 20 µM NF023 for 5 min, 10 µM CPH only decreased the pPKA levels by 2.4±3.7% and 3.1±4.3% (*n* = 6), respectively, which is significantly different from the decrease seen with CPH alone (24.5±6.1%, Fig. 5D).

**Figure 6:**
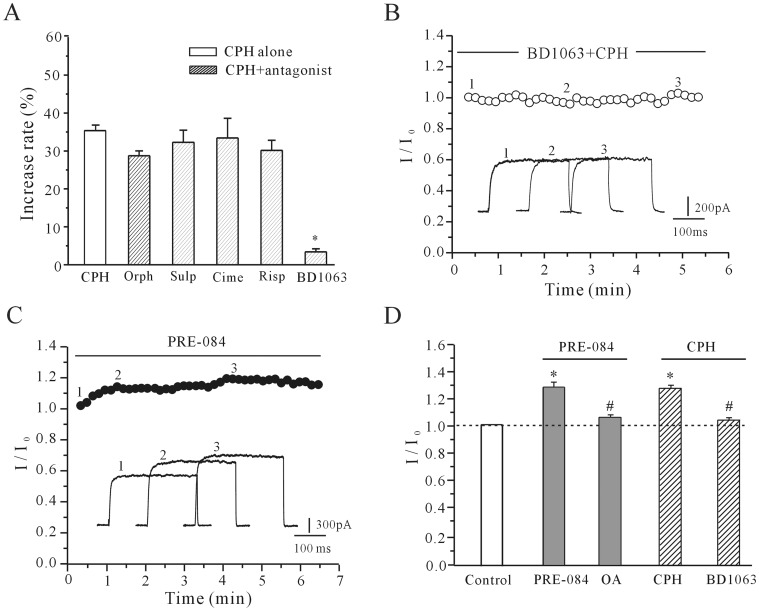
The effect of G_i_ protein-coupled receptor antagonists and sigma-1 receptor antagonists/agonists on the CPH-induced increase in the *I*_K_. (A) The percentage of the *I*_K_ increase that is induced by CPH in the presence of orphenadrine (Orph), sulpiride (Sulp), cimetidine (Cime), risperidone (Risp), and BD1063. (B) The time course of the *I*_K_ changes induced by CPH in the presence of BD1063. (C) The time course of the *I*_K_ enhancement induced by PRE-084, which is a sigma-1 receptor agonist. (D) A quantitative statistical analysis was conducted to verify the effects of okadaic acid (OA) on the PRE-084-induced *I*_K_ effect and BD1063 on the CPH-induced *I*_K_ effect. The data are reported as the mean ± S.E.M. from 7–10 cells. *P<0.05 when compared with the control; ^#^ *P*<0.05 when compared with CPH and PRE-084 alone.

Although we applied CPH intracellularly to avoid any receptor-dependent effects, CPH is a lipid-soluble and membrane-permeable molecule and has been used as a serotonin and H_1_-histamine receptor antagonist [17]. We hypothesized that G_i_ protein-coupled receptors might be involved in the CPH-induced increase in the *I*_K_. As shown in Fig. 6A, we administered commonly used G_i_ anatgonists to test this hypothesis. Antagonists of serotonin receptors (risperidone), D_2_-dopamine receptors (sulpiride), muscarinic receptors, H_1_-histamine receptors (orphenadrine) and H_2_-histamine receptors (cimetidine) did not eliminate the CPH-induced *I*_K_ increase. In the presence of risperidone (20 µM), sulpiride (20 µM), orphenadrine (50 µM) and cimetidine (20 µM), 10 µM CPH increased the *I*_K_ by 33.41±5.19% (*n* = 5),32.27±3.2% (*n* = 6), 28.74±1.28% (*n* = 6) and 30.14±2.67% (*n* = 5), respectively, which is similar to the effect seen with CPH alone (36.1±2.01%, Fig. 6A) However, when cortical neurons were incubated with a sigma-1 receptor antagonist (BD1063), the effect of CPH was abolished. The addition of 20 µM BD1063 reduced the 10 µM CPH-induced increase in the *I*_K_ to 3.4±0.8% (*n* = 9), which indicated that the sigma-1 receptor might be involved (Fig. 6A).

Previous studies have indicated that the sigma-1 receptor is widely distributed in CNS neurons and is associated with multiple intracellular signaling pathways [11]. Therefore, a specific agonist and an antagonist of the sigma-1 receptor were used to explore whether the intracellular application of CPH would activate the sigma-1 receptor and induce an increase in the *I*_K_. When the sigma-1 receptor was blocked by 20 µM BD1063, the CPH-induced increase in the *I*_K_ was reduced to 3.4±0.8% (*n* = 9) (Fig. 6B). In contrast, the sigma-1 receptor agonist PRE-084 mimicked the effect of CPH on the *I*_K_ and elicited a gradual increase in the *I*_K_ amplitude (Fig. 6C). The application of 20 µM PRE-084 increased the *I*_K_ by 26.8±3.7% (*n* = 9), which is similar to the effect produced by CPH (Fig. 6D). Similar with the observation for CPH, okadaic acid also reduced PRE-084-induced *I*_K_ increase (Fig. 6D). In the presence of 200 nM okadaic acid, 20 uM PRE-084 only increase *I*_K_ by 6.94±0.92% (n = 6).

**Figure 7:**
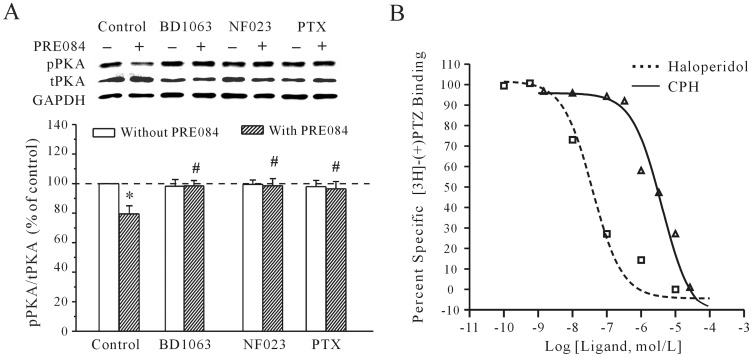
The effect of sigma-1 receptor agonist on the pPKA level and the affinity of CPH for the sigma-1 receptor. (A) pPKA levels in response to the application of either a sigma-1 receptor agonist (PRE-084) or PRE-084 along with a sigma-1 receptor antagonist (BD1063). PTX and NF023 reversed the PRE-084**-**induced decrease in pPKA. The data are reported as the mean ± S.E.M. from 4 independent experiments. *P<0.05 when compared with the control; ^#^ *P*<0.05 when compared with PRE-084 alone. (B) The competitive binding curves of CPH and haloperidol against the radioactive sigma-1 receptor ligand [^3^H]-(+)-pentazocine (PTZ). All of the values are reported as the mean ± S.E.M. of four independent experiments.

We also measured the effect of a sigma-1 receptor agonist and antagonist on the activity of pPKA. Consistent with the *I*_K_ recording, activating the sigma-1 receptor with PRE-084 induced a significant decrease in the intracellular levels of pPKA, and blocking the sigma-1 receptor with BD1063 significantly suppressed the PRE-084-induced decrease of pPKA levels by 4.1±3.2% (*n* = 6). Moreover, blocking G_i_ with PTX and NF023 significantly reduced the inhibitory effect of PRE-084 on pPKA levels (Fig. 7A). After the cortical neurons were incubated with 200 ng/ml PTX for 2 hours or 20 µM NF023 for 5 min, 20 µM PRE-084 only decreased the pPKA levels by 5.41±3.3% and 3.6±2.7% (*n* = 6), respectively. This result was similar to that obtained by the application of CPH alone.

To further confirm that the CPH-induced increase of the *I*_K_ was associated with the sigma-1 receptor, the affinity of CPH for the sigma-1 receptor was determined by a competitive-binding assay. Sigma-1 receptors were labeled in rat liver homogenates using the radioactive sigma-1 receptor ligand [^3^H]-(+)-pentazocine (5 nM). Figure 7C shows the competitive binding curves of CPH and haloperidol against [^3^H]-(+)-pentazocine. The IC_50_ value (50% inhibition of the specific binding of [^3^H]-(+)-pentazocine) for CPH was 2.49±0.63 µM, which is in line with the concentration used in the electrophysiology and PKA activity assays. The dose-response curve showed that CPH interacted with the sigma-1 receptor in a competitive manner. Furthermore, the results also showed that the K*_i_* of CPH was 0.93±0.23 µM, which is consistent with other sigma-1 receptor ligands [10], [11]. The K*_i_* values were derived from the IC_50_ values according to the equation K*_i_*  =  *IC*_50_/(1+ C/Kd) [18], where C is the concentration of the radioligand [^3^H]-(+)-pentazocine (5 nM) and Kd (3 nM) is the dissociation constant of the corresponding [^3^H]-(+)-pentazocine binding to the sigma-1 receptor in rat liver homogenates [18].

**Figure 8:**
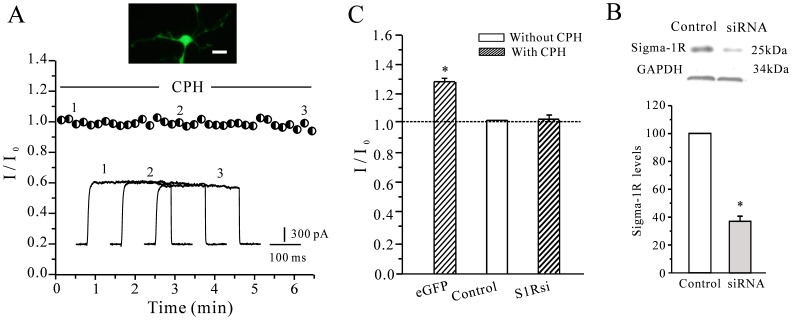
The effect of knocking down the sigma-1 on the CPH-induced increase of the *I*_K_. (A) Typical current recordings from cortical neurons that were transfected with sigma-1 receptor siRNAs after the intracellular application of 10 µM CPH. The scale bar represents 10 µm. (B) A statistical analysis of the effects of knocking down sigma-1 receptor (S1Rsi) expression on the CPH-induced *I*_K_ enhancement. eGFP means the cells transfected with eGFP vectors as control. The data are reported as the mean ± S.E.M. from 7–10 cells. *P<0.05 when compared with the control. (C) A Western blot was used to assess the efficiency of the sigma-1 receptor siRNA in HEK-293 cells that endogenously express the sigma-1 receptor. The data are reported as the mean ± S.E.M. from 4 different experiments. *P<0.05 when compared with the control).

We further investigated whether the CPH-induced increase in the *I*_K_ was dependent on the sigma-1 by knocking down the expression of sigma-1 receptor in cortical neurons with interfering RNAs (siRNAs). A plasmid containing a specific sigma-1 receptor siRNA along with enhanced green fluorescent protein was transfected into the cultured mouse cortical neurons. Green fluorescence was observed 48 h after transfection. To avoid a background effect of the transfection process on the current amplitude and CPH, a group of cells that had been transfected with a random sequence and EGFP were used as a negative control. Figures 8A show the typical current recordings in a cortical neuron transfected with siRNA against sigma-1 receptor. When the sigma-1 receptor expression was knocked down, the effect of CPH on the *I*_K_ was almost eliminated, and the addition of 10 µM CPH only increased the *I*_K_ by 6.09±1.7% (*n* = 8). The CPH-induced increase of the *I*_K_ recorded from the eGFP control group was similar to that of the untransfected cells. A statistical analysis of these data is shown in Fig. 8B. Concurrently, the efficiency of the siRNA in HEK-293 cells that endogenously express the sigma-1 receptor was also analyzed. A Western blot showed that siRNAs against the sigma-1 significantly inhibited the level of protein that was expressed in the HEK-293 cells (Figs. 8C). This result suggested that both the sigma-1 receptor is needed to see the effect of CPH on the *I*_K_.

### The Kv2.1 α-subunit is Involved in the Cyproheptadine-induced *I*_K_ Increase

**Figure 9:**
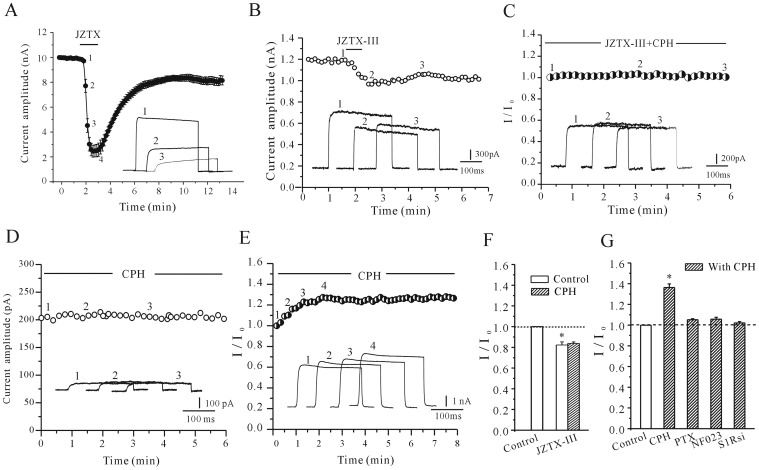
The Kv2.1 α-subunit is implicated in the CPH-induced increase of the *I*_K_ in both cortical neurons and HEK-293 cells transfected with the Kv2.1 α-subunit. (A) The effect of 100 nM JZTX-III on current recordings from HEK-293 cells transfected with the Kv2.1 α-subunit. (B) The time course of the changes in the *I*_K_ amplitude in cortical neurons treated with 100 nM JZTX-III. (C) The effect of intracellular 10 µM CPH on the *I*_K_ in the present of JZTX-III in the bath solution. (D) The effect of intracellular 10 µM CPH on the K^+^ current recordings from HEK-293 cells transfected with eGFP vectors alone. (E) The effect of intracellular 10 µM CPH on the K^+^ current recordings from HEK-293 cells transfected with Kv2.1 α-subunit. (F) A statistical analysis of the effects of 10 µM CPH on the cortical neurons *I*_K_ in the present of JZTX-III. (G) A statistical analysis of the effects of JZTX-III, PTX, NF023, sigma-1 receptor knockdown (Sigma-1Rsi) on the CPH-induced increase in the Kv2.1 current. The data are reported as the mean ± S.E.M. from 8–12 cells. *P<0.05 when compared with the controls; ^#^ *P*<0.05 when compared with CPH applied to cortical neurons.

It has been previously reported that the Kv2.1 α-subunit, which is expressed at high levels in most mammalian CNS neurons, is a major contributor to the *I*_K_ channels and plays a crucial role in regulating neuronal excitability [19]. To address the potential role of Kv2.1 in the CPH-induced increase of *I*_K_ channel activity, Jingzhaotoxin-III (JZTX-III), which has been previously demonstrated to specifically inhibit the Kv2.1 α-subunit in HEK-293 cells, was used [20]. 100 nM JZTX-III significantly inhibited 87.2%±2.12% (n = 5) of the Kv2.1 transfected HEK293 cells (Fig. 9A). An inhibition of the *I*_K_ amplitudes by 28.5±4.4% (*n* = 5) was also observed in primary cortical neurons after 100 nM JZTX-III was applied to the bath solution (Fig. 9B). In the presence of 100 nM JZTX-III, the increase in the *I*_K_ that was induced by CPH decreased to 8.2±3.9% (*n* = 7), which indicates that the elimination of Kv2.1 activity prevents the CPH-induced increase of the *I*_K_ in cortical neuron (Fig. 9C and 9F). Using HEK-293 cells, which endogenously express the sigma-1 receptor and were transfected with the exogenous Kv2.1 α-subunit, the experiments that had already been performed in cortical neurons were repeated. As the control, 10 µM CPH did not affect the small endogenous background current of HEK293 cells which was only transfected with EGFP (Fig. 9D). However, 10 µM CPH significantly increased the Kv2.1 currents in the HEK-293 cells by 36.32±3.43% (Fig. 9E and 9G, *n* = 12). Furthermore, when G_i_ was blocked by 200 ng/ml PTX or 20 µM NF023, the application of 10 µM CPH only increased the Kv2.1 currents by 5.24±0.98% (*n* = 10) and 4.31±1.18% (*n* = 6), respectively. When both the expression of the Kv2.1 α-subunit and either the sigma-1 receptor or the was knocked down in the HEK-293 cells, the CPH-induced increase in Kv2.1 currents was reduced to 5.46±1.32% (*n* = 5) and 5.68±0.47% (*n* = 8), respectively. A statistical analysis of these data is shown in Fig. 9G.

## Discussion

Recent studies have examined the effects of CPH independent of its function as a histamine and serotonin receptor antagonist [7], [9]. In this study, we investigated the effects of CPH on the *I*_K_, and the underlying receptor or signal mechanism involved in its effects on the *I*_K_. We demonstrated for the first time that CPH enhances the *I*_K_ in cultured cortical neurons through a G_i_-dependent cAMP/PKA pathway via activation of the sigma-1 receptor.

Ion channels as membrane proteins can be modulated through direct protein-protein interactions or by indirect, membrane-bound, receptor-mediated phosphorylation or dephosphorylation of the channels. Previous studies have shown that CPH directly inhibits K^+^, Na^+^ and Ca^2+^ channels in cardiac cells [6]. In this study, PTX treatment reduced *I*_K_ responses to CPH, and cells perfused internally with NF023, which is known to be a very potent inhibitor of G_i_ protein-dependent processes [21], eliminated the CPH-induced increase in the *I*_K_. Likewise, either blocking constitutive phosphorylation of *I*_K_ channel with the PP1 inhibitor [22] or inhibiting PKA activity with Rp-cAMP interfered with the CPH-induced increase in *I*_K_. Moreover, the measured changes in PKA activity suggest that CPH inhibits the cAMP/PKA pathway. These findings therefore suggest a novel mechanism by which CPH increases voltage-dependent K^+^ channels in cortical neurons that involves both G*_i_* protein-coupled receptors and cAMP/PKA inhibition. Since CPH did not alter the gating property of *I*_K_, increase of *I*_K_ might caused by an increase in current density or channel opening rate.

CPH possesses multiple pharmacological activities due to its relatively high affinity for various receptors, including serotonergic, dopaminergic, histaminergic, adrenergic and muscarinic receptors [23]. In this study, we first attempted to avoid the serotonin, dopamine, or histamine receptor-dependent effects by applying CPH intracellularly. In addition, our results demonstrated that the specific receptor antagonists (against serotonin, dopamine, histamine and muscarinic receptors) that are associated with CPH’s known activity as well as the G_i_ protein-coupled receptor antagonists all failed to interfere with the CPH-induced increase of the *I*_K_. In contrast, applying a sigma-1 receptor antagonist or knocking down the sigma-1 receptor in cortical neurons eliminated the effect of CPH on the *I*_K_. Interestingly, the competitive binding assay using the liver tissues which express high abundant of sigma-1 receptor [24] showed that CPH can act as a ligand for the sigma-1 receptor. In our experimental binding system, the K*_i_* of haloperidol for the sigma-1 receptor was similar to the results reported by Klouz et al [18]. The sigma-1 receptor is widely expressed in the CNS and is present at high levels in the cortex, the hippocampus and the striatum [25]. It binds to a vast number of synthetic compounds whose features include a common N-substituted trace amine [11]. Because CPH also has this N-substituted trace amine, and the sigma-1 receptor binding site is located intracellularly [10], it is highly likely that intracellular CPH might rapidly bind to the sigma-1 receptor.

Given sigma receptors and ion channels are distinct proteins and the cloned sigma-1 receptor does not have the typical structure of a G protein-coupled receptor [26], a relay mechanism will be required to transmit the signal of the target ion channels when the sigma-1 is activated. Earlier studies in melanotroph cells have demonstrated a Gs-dependent modulation of Kv channels by sigma receptors [27], but whether G protein-coupled receptor is involved or not remain unknown. Kim et al. have recently demonstrated a physical and functional association of the sigma-1 receptor with the µ-opioid receptor [28]. They also established that the sigma-1 receptor can directly associate with the µ-opioid receptor and that this association allows selective antagonists of the sigma-1 receptor to potentiate µ-opioid receptor-induced cell signaling. Coincidentally, the µ-opioid receptor is a heterotrimeric G protein-coupled receptor, and µ-opioid receptor activation has been shown to inhibit the cAMP/PKA pathway by G_i_ activation [29]. In this study, we also experimentally observed that knocking down µ-opioid receptor expression in cortical neurons interfered with the CPH-sigma-1 receptor-mediated increase in the *I*_K_. (data not shown) Moreover, activating µ-opioid receptor with agonist mimicked the effect of CPH on the *I*_K_ while the antagonist of µ-opioid receptor failed (data not shown). It is thus highly likely that both the sigma-1 receptor and the µ-opioid receptor were needed to see the effect of CPH on the *I*_K_ of cortical neurons. However, further study is needed for conforming whether both of sigma-1 and µ-opioid receptor was functional coupled in cortical neuron, and CPH as sigma-1 ligand in modulating the µ-opioid receptor-induced signaling.

In most studies, the modulation of voltage-gated K^+^ and Ca^2+^ channels by the sigma-1 receptor does not involve transduction mechanisms, such as G protein signaling or phosphorylation; instead, a direct interaction between the two proteins has been suggested [30]–[32]. In support of this idea, both Kv1.4 and the sigma-1 receptor can be coimmunoprecipitated from membrane lysates that have been prepared from both rat posterior pituitary cells and mRNA-injected Xenopus oocytes. However, the effect of sigma-1 receptor ligands on voltage-dependent ion channels has been inhibitory in almost all of these previous reports; additionally, most of these studies have been conducted in non-neuronal cells or in Xenopus oocytes [11], [30], [33]. In contrast, our study found that CPH enhanced the *I*_K_ via the activation of the sigma-1 receptor and the µ-opioid receptor and the inhibition of cAMP/PKA pathway, which is different from previous reports. This discrepancy might result from the cell type tested due to the presence or absence of the endogenous µ-opioid receptor. It also might be due to the types of K^+^ channels that were studied. Native neuronal *I*_K_ channels are mainly composed of members of the Kv2 subfamily, which are easily modified by the cAMP/PKA pathway through phosphorylation or dephosphorylation mechanisms [34]–[36]. This process may explain why the CPH-induced activation of the sigma-1 receptor and the subsequent modulation of the *I*_K_ in cortical neurons were associated with an indirect mechanism involving the activation of both the µ-opioid receptor/G_i_-protein and the cAMP/PKA pathway in this study. In addition to the µ-opioid receptor, previous studies by Mavlyutov et al. showed colocalization of both the Kv2.1 potassium channels and the muscarinic m2AChR with the sigma-1 receptor in mouse motoneurones [37], [38]. The resolution of the data, as pointed out by Mavlyutov et al., is limited and the detected Kv2.1 potassium channels, the muscarinic m2AChR and the sigma-1 receptor may lie in different membranes. Further functional identification of their direct interactions is warranted.

Electrophysiological studies have revealed that most mammalian neurons express multiple types of voltage-gated K^+^ channels with distinct time- and voltage-dependent properties. The Kv2 (Shab) genes in many species encode components of potassium channels with sustained currents. In particular, the Kv2.1 subunit, which is highly expressed in most mammalian CNS neurons, is a major contributor to the *I*_K_ channels and plays a crucial role in regulating neuronal excitability. Previous studies and recent works from our laboratory have confirmed by that Kv2.1 is a major component of cortical neuronal *I*_K_ channels.

In this study, we also reported that CPH might target the Kv2.1 α-subunit, as revealed by the use of a specific toxin (JZTX-III) that has been reported to selectively block Kv2.1 α-subunits [39]. We further ascertained the specificity in a heterologous system using HEK-293 cells, which endogenously express the sigma-1 receptor [11] and the µ-opioid receptor [7], by transfecting an expression vector containing the Kv2.1 α-subunit. The results were similar to those observed in the primary cortical neurons as we observed increased Kv2.1 currents by CPH in the HEK-293 cells transfected with the Kv2.1 α-subunit. CPH, however, did not affect Kv2.2 currents in transfected HEK-293 cells (data not shown). Moreover, CPH-induced enhancement of the Kv2.1 channels in transfected HEK-293 cells was also eliminated after blocking G_i_ proteins with PTX and NF023 or knocking down the expression of the sigma-1 receptors. The overall results not only indicated that the Kv2.1 α-subunits were primarily affected by CPH but also supported the idea that CPH enhanced the *I*_K_ via the activation of the sigma-1 receptor/G_i_ protein and cAMP/PKA pathways.

Although previous studies have demonstrated that CPH modulates membrane ion channels in the kidney and cardiovascular system [6], there have been few studies examining the influence of CPH on voltage-gated K^+^ currents in the nervous system. Because voltage-gated K^+^ channels play a crucial role in maintaining the resting membrane potential of the neuron as well as the duration and frequency of action potentials, modulators of these channels would likely have therapeutic effects in various neurological and psychological disorders [40]. For example, K_V_ channel blockers are expected to restore normal firing in depression and cognitive dysfunction, while K_V_ channel activators should be useful in reducing the pathological hyperexcitability that occurs in epilepsy [41]. In addition, because the sigma-1 receptor has been linked to the modulation of processes involving memory and learning, depression, and psychosis [42], CPH’s association with the sigma-1 receptor and the subsequent increase in *I*_K_ channel activity may contribute to its apparent clinical efficacy as an antidepressant and an antipsychotic.

**Figure 10:**
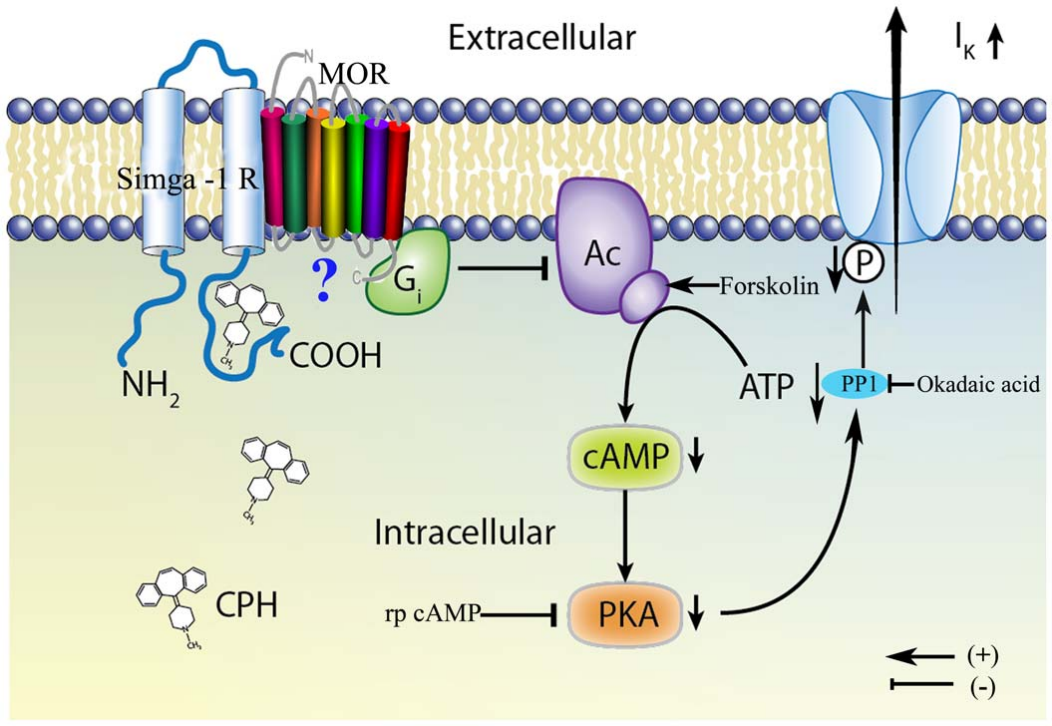
A proposed model depicting the mechanisms involved in the increase of the *I*_K_ by intracellular CPH in mouse cortical neurons. CPH interacts with the sigma-1 receptor at an intracellular biding site and associates with the µ-opioid receptor, which results in an increase in the *I*_K_ via G_i_ and the cAMP/PKA pathway.

A proposed model illustrating the signaling cascade that might be associated with CPH in cortical neurons is shown in Fig. 10. We propose that CPH, acting through its association with the sigma-1 receptors at an intracellular binding site, increases the *I*_K_ via G_i_ activity and cAMP/PKA pathway inhibition.

## Materials and Methods

### Ethics Statement

This study was carried out in strict accordance with the recommendations in the Guide for the Care and Use of Laboratory Animals of the National Institutes of Health. The protocol was approved by the Committee on the Ethics of Animal Experiments of the Fudan University (Permit Number: 2007-0002). All surgery was performed under sodium pentobarbital anesthesia, and all efforts were made to minimize suffering.

### Cultures of Mouse Cortical Neurons

ICR (Swiss Hauschka) mice were purchased from the Shanghai Laboratory Animal Center at the Chinese Academy of Sciences (Shanghai, China). Primary neuronal cultures were prepared from the cerebral neocortex of 15-day-old embryonic mice as originally described. Briefly, the cerebral neocortices were dissected from embryonic ICR mice. The dissociated cortical neurons were then plated onto poly-L-lysine-coated (10 µg/ml) 35-mm dishes (Costar, Corning, NY) at a density of 1×10^5^ cells per dish. The cultured cells were incubated at 37°C with 5% CO_2_ in Dulbecco’s Modified Eagle’s Medium (DMEM) supplemented with 10% fetal calf serum, 10% heat-inactivated horse serum and a 1% antibiotic-antimycotic solution. After 24 hours in culture, cytosine 1-β-D-arabinofuranoside (5 µM) was applied to the culture medium for 48 hours to eliminate actively proliferating fibroblasts. On day 4, the cultured cells were washed and maintained in fresh medium. All of the experiments were conducted with cortical neurons that had been in culture for 6–10 days.

### Patch-clamp Recordings

Whole-cell currents were recorded in cortical neurons using a patch-clamp technique. Prior to the *I*_K_ recordings, the culture medium was replaced with a bath solution containing 140 mM NaCl, 2.5 mM KCl, 10 mM HEPES, 1 mM MgCl_2_, and 0.001 mM TTX. The pH was adjusted to 7.4 using NaOH. Soft glass recording pipettes were filled with an internal solution containing 135 mM K gluconate, 10 mM KCl, 10 mM HEPES, 1 mM CaCl_2_, 2 mM MgCl_2_, 2 mM ATP·Mg, 0.1 mM GTP·Na_3_, and 10 mM EGTA. The pH of this solution was adjusted to 7.3 using KOH. The pipette resistance was 5–7 MΩ after it was filled with the internal solution. All of the recordings were performed at room temperature (23–25°C).

### DNA Constructs and Cell Transfection

Total RNA was isolated from the primary cultures of mouse cortical neurons using a commercial kit according to the manufacturer’s instructions (Qiagen Mini RNeasy, Qiagen, Valencia, CA, USA). First-strand synthesis was performed using SuperScript II reverse transcriptase (Invitrogen, Carlsbad, CA, USA). The following primer set was designed to amplify mouse Kv2.1: a forward primer (5′ TGGCTCGAGATGCCGGCGGGCATG 3′) that included an XhoI site and a reverse primer (5′ ATACAGAATTCGGATACTCTGATCCCT 3′) that included an EcoRI site (GenBank NM_008420). Mouse Kv2.1 cDNA was ligated into the pll3.7 vector using the XhoI and EcoRI restriction sites. Each gene was fused to the N-terminus of EGFP, which was used as a fluorescent marker to identify transfected human embryonic kidney (HEK)-293 cells which purchased form the cell bank of Chinese Academy of Science (Shanghai, China). All of the constructs were verified by DNA sequencing. The plasmids were extracted using a Qiagen plasmid midi kit (Qiagen, Valencia, CA, US). The DNA concentration and purity of each plasmid were determined by measuring the absorbances at 260 and 280 nm, respectively. HEK-293 cells were transfected using the calcium phosphate method. The average transfection efficiency was above 80%. Two days after transfection, the HEK-293 cells were examined for green fluorescence.

### RNA Interference Knockdown of Sigma-1 and µ-opioid Receptors

The plasmid used to silence the sigma-1 receptor was constructed using the pGPU6/GFP/Neo siRNA vector (GenePharma, Shanghai, China). An siRNA sequence corresponding to nucleotides 500–519 of the human sigma-1 receptor open reading frame (PubMed nucleotide ID: NM005866), which is identical to the mouse sequence in this region, was inserted into the pGPU6/GFP/Neo vector, and the plasmid was transfected into both primary mouse cortical neurons and mammalian HEK-293 cells. As a negative control, a random siRNA sequence was inserted into the pGPU6/GFP/Neo vector. To silence the µ-opioid receptor gene, a plasmid was constructed using the pll3.7 siRNA vector. The siRNA sequence corresponding to nucleotides 392–409 of the mouse MOR-1 (ID: NM_001039652) was inserted into the pll3.7 vector, and the plasmid was transfected into both cortical neurons and HEK-293 cells. Transfection was accomplished using Lipofectamine 2000 (Invitrogen, Carlsbad, CA, USA) and Opti-mem media (Gibco, Grand Island, NY, USA) according to the manufacturers’ instructions. Sigma-1 receptor and µ-opioid receptor siRNAs were cotransfected with enhanced green fluorescent protein (EGFP) to label the transfected cells. Sigma-1 and µ-opioid receptor protein levels were detected by an immunoblot with antibodies against both proteins. Anti-Sigma-1 receptor antibody was a gift from Dr. Teruo Hayashi’s lab and anti-µ-opioid receptor antibody was purchased from Millipore (Millipore, Billerica, MA).

### Phosphorylated Protein Kinase a Assay

Cells were lysed in HEPES-NP40 lysis buffer (20 mM HEPES, 150 mM NaCl, 0.5% NP-40, 10% glycerol, 2 mM EDTA, 100 µM Na3VO4, 50 mM NaF, pH 7.5, and 1% proteinase inhibitor cocktail) on ice for 30 min [43]. After centrifugation, the supernatant was mixed with 2× sodium dodecyl sulfate loading buffer and boiled for 5 min. The proteins were separated on a 10% polyacrylamide gel, transferred to polyvinylidene difluoride membranes (Millipore, MA USA), blocked with 10% nonfat milk and incubated with mouse monoclonal antibody against PKA (1∶200; Santa Cruz biotechnology Inc., CA, USA), rabbit polyclonal antibody against the pPKA catalytic subunits (1∶1000; Santa Cruz biotechnology Inc., CA, USA) or rabbit monoclonal antibody against GAPDH (1∶1000; Sigma) at 4°C overnight. After extensively washing in TBST, the membrane was incubated with horseradish peroxidase-conjugated anti-mouse or anti-rabbit IgG (1∶10,000) (KangChen Bio-Tech, China) for 2 h at room temperature. Chemiluminescent signals were generated using a SuperSignal West Pico trial kit (Pierce, USA) and detected by using ChemiDoc XRS System (Bio-Rad Laboratories, Inc., CA, USA). The intensity of the protein band was densitometrically quantified by Image Lab (Bio-Rad Laboratories, Inc., CA, USA). The protein measurements were normalized to GAPDH and control/GAPDH as 100%.

### Ligand-receptor Binding Assay

Membranes from Sprague-Dawley rat (150–200 g) livers were prepared for the sigma-1 receptor binding assay using a previously described method [24] with minor modifications. The livers were quickly processed and homogenized with a glass homogenizer (8–10 up and down strokes) in 6 ml/g of cold buffer (0.32 M sucrose in 50 mM Tris, pH 7.4) and then homogenized with a Tissue-Tearor (Biospec Products, Inc.). The cell suspension was centrifuged at 1200 *g* for 10 min at 4°C, and the supernatant was decanted and recentrifuged at 25000 g for 20 min at 4°C. The resulting pellet was then resuspended in 5–6 ml/g of 50 mM Tris (pH 7.4) and centrifuged at 25000 *g* for 20 min at 4°C. This procedure was repeated twice. The pellet was resuspended in a final volume (5–6 ml/g) of buffer and stored at −80°C until use. The membrane preparation contained approximately 2.5 mg protein/ml, as determined by the Lowry method [44].

The affinity of CPH for the sigma-1 receptor was determined by a competitive binding assay. The membrane preparations, which contained approximately 250–400 µg of protein, were incubated in duplicate for 180 min at 30°C with various concentrations of CPH (1 nM–100 µM) and 5 nM [^3^H]-(+)-pentazocine (28 Ci/mmol; Perkin-Elmer, Boston, MA, USA) in a total volume of 200 µl of binding buffer (50 mM Tris and 4 mM MgCl_2_, pH 7.4). The specific binding was assayed in the presence of 10 µM haloperidol. The reaction was stopped by rapid filtration through a Whatman GF/B glass fiber filter and subsequent washing with cold buffer (50 mM Tris and 5 mM EDTA, pH 7.4) using a Brandel 24-well cell harvester. The filters were then dried at 80°C for 30 min in a drying oven and immersed in a scintillation cocktail. The radioactivity of each filter was determined with a MicroBeta liquid scintillation counter. The IC_50_ values (i.e., the concentration of unlabeled test compound needed to cause a 50% inhibition of the specific binding of the radiolabeled ligand) from these assays were calculated by nonlinear regression (PRISM, Graphpad, San Diego, CA, USA) using a sigmoidal function. Inhibition constant (K_i_) values were calculated from the IC_50_ values according to Cheng and Prusoff [45].

### Data Acquisition and Analysis

All of the currents were recorded using an Axopatch 200B amplifier (Axon Instruments, Foster City, CA, USA) that was operated in voltage-clamp mode. A Pentium computer was connected to the recording equipment with a Digidata 1300 analog-to-digital (A/D) interface. The current was digitally sampled at 100 µs (10 kHz). The current signals were filtered by a 5-kHz, five-pole Bessel filter. The currents were corrected online for leak and residual capacitance transients by a P/4 protocol. The data acquisition and analysis were performed with pClamp 8.01 software (Axon Instruments, Foster City, CA, USA) and/or Origin6.1 (MicroCal, Northampton, MA, USA). The statistical analysis was performed using Student’s *t*-test with non-paired or paired comparisons when relevant. The values are given as the mean ± S.E.M. with *n* representing the number of cells tested. A value of *P*<0.05 was considered a significant statistical difference between groups. When multiple comparisons were made, the data were analyzed by a one-way ANOVA.

### Chemicals

Cytosine 1-β-D-arabinofuranoside, tetraethylammonium chloride (TEA), NF023, 2-(4-morpholinethyl)-1-phenylcyclohexanecarboxylate(PRE-084), forskolin, BD1063, cyproheptadine (CPH), tetrodotoxin (TTX), pertussis toxin (PTX), dibutyryl cAMP (db-cAMP), 4-aminopyridine (4-AP), Rp-Cyclic 3′,5′-hydrogen phosphorothioate adenosine triethylammonium salt (Rp-cAMPS), sulpiride, risperidone, orphenadrine, cimetidine, and poly-L-lysine were all purchased from Sigma-Aldrich (St. Louis, MO, USA). The sigma-1 receptor antibody was a gift from Dr. Teruo Hayashi’s laboratory. Fetal calf serum, DMEM and the antibiotic–antimycotic solution were all obtained from Gibco Life Technologies (Grand Island, NY, USA). CPH solutions were prepared immediately before use. CPH was first dissolved in methanol and then diluted in the previously described bath solution with a final methanol concentration <0.1%. This small amount of methanol did not have a significant effect on the K^+^ currents.
